# Novel application of luciferase assay for the *in vitro* functional assessment of *KAL1* variants in three females with septo-optic dysplasia (SOD)

**DOI:** 10.1016/j.mce.2015.09.010

**Published:** 2015-12-05

**Authors:** Mark J. McCabe, Youli Hu, Louise C. Gregory, Carles Gaston-Massuet, Kyriaki S. Alatzoglou, José W. Saldanha, Angelica Gualtieri, Ajay Thankamony, Ieuan Hughes, Sharron Townshend, Juan-Pedro Martinez-Barbera, Pierre-Marc Bouloux, Mehul T. Dattani

**Affiliations:** a Section of Genetics and Epigenetics in Health and Disease, Genetics and Genomic Medicine Programme, UCL Institute of Child Health, London, UK; b Kinghorn Centre for Clinical Genomics, Garvan Institute of Medical Research, Darlinghurst, NSW, Australia; c St Vincent's Clinical School, UNSW Australia, Sydney, NSW, Australia; d Centre for Neuroendocrinology, Royal Free Hospital and University College Medical School, University College London, London, UK; e Department of Anaesthesiology, Nanjing Medical University First Affiliated Hospital, Jiangsu Province Hospital, Nanjing 210029, China; f Neural Development Unit, UCL Institute of Child Health, London, UK; g Centre for Endocrinology, William Harvey Research Institute, Barts and the London School of Medicine and Dentistry, Queen Mary University of London, John Vane Science Centre, Charterhouse Square, London, UK; h Division of Mathematical Biology, National Institute for Medical Research, London, UK; i University of Cambridge, Addenbrookes Hospital, Cambridge, UK; j Princess Margaret Hospital for Children, Subiaco, Western Australia, Australia

**Keywords:** Luciferase assay, Septo-optic dysplasia, *KAL1*, Kallmann syndrome, Females

## Abstract

*KAL1* is implicated in 5% of Kallmann syndrome cases, a disorder which genotypically overlaps with septo-optic dysplasia (SOD). To date, a reporter-based assay to assess the functional consequences of *KAL1* mutations is lacking. We aimed to develop a luciferase assay for novel application to functional assessment of rare *KAL1* mutations detected in a screen of 422 patients with SOD.

Quantitative analysis was performed using L6-myoblasts stably expressing FGFR1, transfected with a luciferase-reporter vector containing elements of the FGF-responsive osteocalcin promoter.

The two variants assayed [p.K185N, p.P291T], were detected in three females with SOD (presenting with optic nerve hypoplasia, midline and pituitary defects). Our novel assay revealed significant decreases in transcriptional activity [p.K185N: 21% (p < 0.01); p.P291T: 40% (p < 0.001)].

Our luciferase-reporter assay, developed for assessment of *KAL1* mutations, determined that two variants in females with hypopituitarism/SOD are loss-of-function; demonstrating that this assay is suitable for quantitative assessment of mutations in this gene.

## Highlights

• We identified variations in X-linked *KAL1* in three girls with septo-optic dysplasia.
• We developed a luciferase assay for novel functional assessment of these variants.
• Variants were loss of function but variably penetrant; other genes may be involved.
• Cellular secretion of one of the mutant KAL1 proteins was partially disrupted.
• We identified *KAL1* expression in developing human pituitary by *in situ* hybridisation.

## 1 Introduction

Septo-optic dysplasia (SOD) is a rare, highly heterogeneous disorder (incidence 1:10,000–1:20,000 live-births) (Patel et al. 2006). The diagnosis is based on the presence of at least two of the following: i) midline defects such as absent septum pellucidum or agenesis of the corpus callosum, ii) optic nerve hypoplasia or iii) anterior pituitary hypoplasia with/without anterior pituitary hormone deficiencies (Kelberman and Dattani, 2007, Webb and Dattani, 2010). The condition is part of a wider spectrum of disorders associated with midline defects of the forebrain and congenital hypopituitarism with phenotypes ranging from cleft palate and SOD, to holoprosencephaly (HPE) and incompatibility with life.

SOD generally occurs sporadically but environmental, epigenetic and genetic factors are implicated in its aetiology. These include mutations in transcription factors that play critical roles in early forebrain and pituitary development, such as *HESX1, SOX2, SOX3*, and *OTX2* (McCabe et al. 2011a). Recently, the scope of potential genetic candidates has widened with the discovery of mutations/variations in genes classically involved in Kallmann syndrome (KS; anosmia/hyposmia associated with hypogonadotrophic hypogonadism) in patients with SOD. These include *Fibroblast Growth Factor Receptor 1* (*FGFR1*), ligand *FGF8* and *PROKR2* (McCabe et al., 2011b, McCabe et al., 2013; Raivio et al. 2012), all implicated in the maturation of gonadotrophin releasing hormone (GnRH) neurons and/or their migration from the olfactory placode to the ventral forebrain (Pitteloud et al., 2007, Falardeau et al., 2008). However, consistent with the overlap in genotypes is the occasional overlap in phenotypes with the SOD spectrum including cleft lip/palate, synkinesia and sensorineural hearing loss among others (Jansen et al., 2000, Massin et al., 2003, Trarbach et al., 2005, Versiani et al., 2007).

The first gene implicated in KS was *KAL1*, which encodes anosmin-1, a large 680 amino acid protein consisting of a whey acidic protein domain, followed by four tandem fibronectin (FnIII) repeats, which is essential for GnRH neuronal migration. Expression in many species is not well characterised, but it is known to be ubiquitously expressed during development and into adulthood in marsupials (Hu et al. 2011). *KAL1* localises to Xp.22.3 in humans and because of this, more males than females were previously screened for mutations in *KAL1* (Söderlund et al. 2002). Recently however, female KS patients have also tested positive for mutations in *KAL1* (Shaw et al. 2011) and this may be due to the location of the gene within the pseudoautosomal region of the X-chromosome, where it may escape X-inactivation (Shaw et al. 2011).

Our previous work identifying mutations/variations in *FGFR1*, *FGF8* and *PROKR2* in large cohorts of patients with congenital hypopituitarism and associated phenotypes including SOD (McCabe et al. 2011b, 2013; Raivio et al., 2012, McCabe et al., 2013) was aided by animal models and functional assays. Frustrating efforts to analyse *KAL1* variants, a gene implicated in 5% of KS cases, is the lack of a suitable animal model (this gene has not been detected in mice) or a luciferase-reporter assay.

We aimed to screen our original cohort of 422 patients with SOD/congenital hypopituitarism, and develop a luciferase-reporter assay for novel functional assessment of detected *KAL1* variants, using a reporter driven by an FGF-responsive osteocalcin promoter (Kim et al. 2003). The promoter is stimulated by the Protein Kinase C pathway which is induced upon the interaction of FGF2 ligand with FGFR1 (Kim et al. 2003). Anosmin-1 is known to enhance activity particularly in the presence of heparan sulphate (HS), and we hypothesised that these interactions would be measurable, and any changes in function due to mutated anosmin-1 discernible (Pitteloud et al., 2007, Falardeau et al., 2008, Hu et al., 2009). In order to gain insight into a role for *KAL1* in the aetiology of congenital hypopituitarism, we also investigated by expression analysis, a role for this gene in human embryonic hypothalamo-pituitary development.

## 2 Materials and methods

### 2.1 Patients

422 patients had previously been recruited from national (n = 325) and international (n = 97) centres between 1998 and 2010. All had midline defects, either SOD and its variants (n = 375, 89%) or HPE and midline clefts (n = 47, 11%) and had previously screened negative for other KS and SOD genes including *FGF8*, *FGFR1*, *PROKR2*, *PROK2*, *CHD7*, *WDR11*, *NELF*, *SOX3*, *SHH* and *HESX1*. Ethical committee approval was granted by the UCL Institute of Child Health/Great Ormond Street Hospital for Children Joint Research Ethics Committee (London, UK) and written informed consent was obtained from the patients and/or parents.

### 2.2 Mutation/variation analysis

The entire coding region of human *KAL1* (NM_000216) was amplified by PCR [35 cycles (details/conditions provided in Table 1)]. Amplified DNA was sequenced using BigDye v1.1 sequencing chemistry (Applied Biosystems) and analysed on a 3730X1 DNA Analyzer (Applied Biosystems/Hitachi, Japan). Variations were compared for conservation across multiple species, screened across 480 ethnically-matched controls and cross-checked against the dbSNP, Exome Sequencing Project (ESP), 1000 genomes and Aggregation Consortium (ExAC; >61,000 genomes) databases.

### 2.3 Protein-prediction modelling of KAL1 variants

The human anosmin-1 sequence (Uniprot AC: P23352; ID: KALM_HUMAN) was used to search the Protein Data Bank (Berman et al. 2000) using PSI-BLAST (Altschul et al. 1997) with default parameters. The top hit reported by the PSI-BLAST run was the solution structure of the extracellular matrix protein anosmin-1 (PDB ID: 1ZLG) (Hu et al. 2005) with a 99% identity between the sequences. This solution structure of the recombinant protein was determined by X-ray scattering and analytical ultracentrifugation, and thus the PDB file only contained alpha-carbon coordinates. In order to generate the main-chain and side-chain coordinates, the program MODELLER (Sali and Blundell, 1993) was used with intermediate refinement. Subsequently, side-chain packing was re-optimised using SCWRL4 (Krivov et al. 2009). Detected variations were modelled using the molecular graphics program Molecular Operating Environment (MOE, 2012) 2012.10; which was also used to analyse the model and generate images.

### 2.4 Functional studies

#### 2.4.1 Constructs and site-directed mutagenesis

Full length wild-type (wt) human *KAL1* had previously been cloned into the pEGFP-N1 expression vector, (BD Biosciences Clontech), which, in the presence of a mutated *KAL1* stop codon, expresses anosmin-1 conjugated to GFP at the C-terminal (Hu et al. 2009). Detected mutations/variations were introduced using the QuikChange II XL Site-Directed Mutagenesis Kit (Stratagene) according to manufacturer's directions; success of mutagenesis was confirmed by direct sequencing as detailed earlier.

#### 2.4.2 Cell culture

Cos7 cells (American Type Culture Collection, ATCC, Manassas, VA, USA) and *FGFR1*-stably transfected L6-myoblast cells (kindly provided by Professor Erhard Hohenester, Imperial College, London, UK) were maintained in a humidified CO_2_ (5%) incubator at 37 °C in Dulbecco's Minimum Essential Medium (DMEM) supplemented with 10% fetal bovine serum (FBS, Gibco, Grand Island, NY, USA). Media was replaced at 2-day intervals.

#### 2.4.3 Transfection of constructs for qualitative analysis

Cos7 cells in 6-well plates were transiently transfected with equal amounts (1 μg) of wt and mutant/variant pEGFP-N1-*KAL1* constructs. Empty pEGFP-N1 (1 μg) vector served as the control. Transfection reagent FuGene6 (Roche) was employed at a 3:1 ratio with the total mass of DNA (1 μg) transfected. Cells were then left for 48 h in the presence of 10% fetal bovine serum prior to fixation for immunocytochemistry or lysis for western blot analysis. Cultures and treatments were performed three times in triplicate.

#### 2.4.4 Transfection of constructs for quantitative analysis

We sought to exploit the known interactions between anosmin-1, heparin and the FGFR1 signalling pathway in a similar manner to that published for FGF ligands (Pitteloud et al., 2007, Falardeau et al., 2008, Hu et al., 2009), in L6-myoblast cells. In contrast to Cos7 cells, L6-myoblasts have low levels of endogenous FGF ligands and receptors (Pitteloud et al. 2007). As mentioned above, we were able to obtain L6-myoblasts which stably express *FGFR1*.

**Supplementary Fig. 1:**
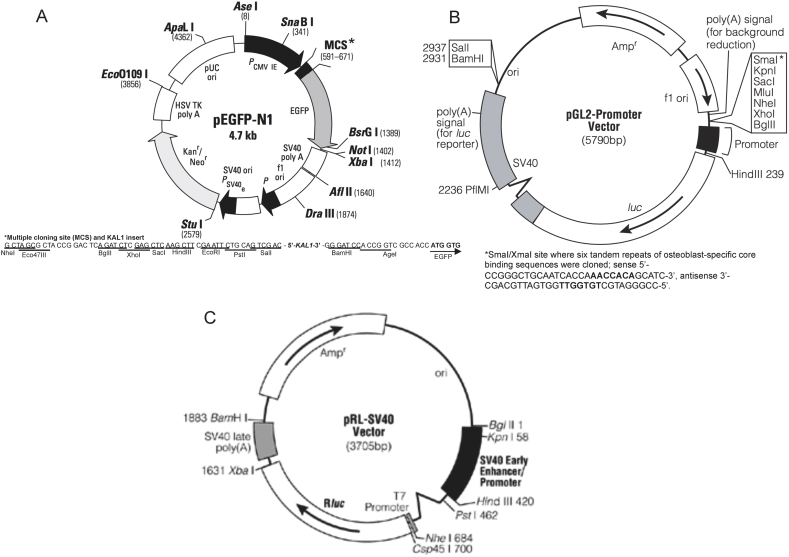
Schematic representation of expression and reporter vectors used for novel application of our luciferase reporter assay for functional assessment of *KAL1* variants *in vitro*. A, Full length human *KAL1* (NM_000216) had previously been cloned into the SalI (5′) and BamHI (3′) restriction sites of the pEGFP-N1 expression vector (Clontech) (Hu et al., 2009). In the presence of the mutated *KAL1* stop codon, the encoded protein anosmin-1 is conjugated to GFP at the C-terminal. Empty pEGFP-N1 vector which expresses GFP alone was used as control. B, The p6xOSE2-Luc reporter vector was produced as previously described (Kim et al., 2003, Park et al., 2010). Six identical osteoblast-specific core binding sequences (bold) (OSE2) of the FGF responsive osteocalcin promoter were synthesised with XmaI overhangs at each 5′ end of the oligonucleotides and ligated. Multimers were then cloned into the SmaI/XmaI restriction site of the pGL2-promoter reporter vector (Promega). C, Luciferase activity stimulated by the *KAL1* expression vector via the p6xOSE2-Luc reporter vector was normalised to the constitutively active Renilla luciferase reporter pRL-SV40 (Promega).

Cells were plated at 1 × 10^5^/well in a 6 well plate. One day before transfection, media was replaced with serum-free medium. Transfection was again conducted with FuGene6 at a 3:1 ratio with DNA (total 2.128 μg) with empty vector or wt/variant *KAL1* constructs (1 μg, as above), in addition to luciferase reporter p6xOSE2-Luc (1 μg) which contains 6 tandem repeats of the osteoblast-specific core binding sequences of the FGF responsive osteocalcin promoter cloned into the XmaI site of the pGL2 promoter reporter vector [Promega; kindly produced and donated by Dr Hyun-Mo Ryoo, School of Dentistry, Seoul National University, South Korea (Kim et al., 2003, Park et al., 2010)]. The efficiency of transfection was standardised to a constitutively active pRL-SV40 Renilla luciferase (Promega) internal control (128 ng). Cells were then incubated for 48 h in the absence of serum. Halfway through this period, cells were treated with 1 nM recombinant human (rh)-FGF2 (PeproTech) with or without 1 μg/ml porcine intestinal mucosa heparin [a type of heparan sulphate (HS); Sigma]. At the end of the culture, cells were lysed, processed and assayed for luciferase activity using the Dual-Luciferase Reporter Assay System (Promega) and read on the FluorStar Optima (BMG technologies). Schematic diagrams of expression and reporter vectors used herein are provided in Supplementary Fig. 1.

### 2.5 Immunocytochemistry

Immunocytochemistry was conducted on paraformaldehyde-fixed Cos7 cells 2 days after wt/variant anosmin-1 transfection to assess localisation. Given that anosmin-1 was conjugated to GFP, no direct immunocytochemical procedures were required to detect the protein. However, in order to assess the localisation of the protein with respect to the cell nuclei and cell membranes, we probed β-catenin of the adherens junction [mouse anti-β-catenin primary antibody (0.5 μg/ml; BD Transduction Laboratories) and goat anti-mouse, Alexa-546 conjugated secondary antibody (1:400; Molecular Probes)] and used nuclear-counterstain 4′,6-diamidino-2-phenylindole dihydrochloride (DAPI; Molecular Probes). Experiments were conducted in duplicates with multiple, representative photographs acquired (n = 3) by epifluorescence microscopy (Nikon Eclipse TE 200).

### 2.6 Western blot analysis

Equivalent amounts of protein extracted from Cos7 cells were separated by SDS-PAGE on 10% gels and transferred onto Amersham Hybond-P, poly (vinylidene difluoride) membranes (GE Healthcare) for Western blot analysis. GFP-anosmin-1 was probed with chick anti-GFP primary antibody (AbCam; ab13970; 1:5000) and goat-anti chicken IgY, HRP-conjugated secondary antibody (AbCam: ab6877; 1:5000). Bands were visualised using ECL (Western blotting detection reagents) chemicals (GE Healthcare) and Amersham Hyperfilm (GE Healthcare) which had been exposed to the membranes.

### 2.7 *In situ* hybridisation

In order to gain some insight into a potential role of *KAL1* in hypothalamo-pituitary development, we obtained human embryos at 6 and 8 weeks of gestation from the Human Developmental Biology Resource (University College London Institute of Child Health, London, UK). An ∼810bp fragment was cloned from the full length wt pEGFP-N1-*KAL1* construct and inserted into pBluescript. *In situ* hybridisation was then conducted on paraffin embedded sections as previously described (Gaston-Massuet et al. 2008).

### 2.8 Statistical analyses

Culture experiments were tested for normal distribution and equal variance before being compared to controls by ANOVA, followed by Student Newman–Keuls test to determine where any differences lay. p < 0.05 values were considered statistically significant. Analyses were performed using SigmaStat version 3.5 (Systat Software, Inc., San Jose, CA) and data have been expressed as mean ± SD.

## 3 Results

### 3.1 Patients and phenotypes

**Fig. 1:**
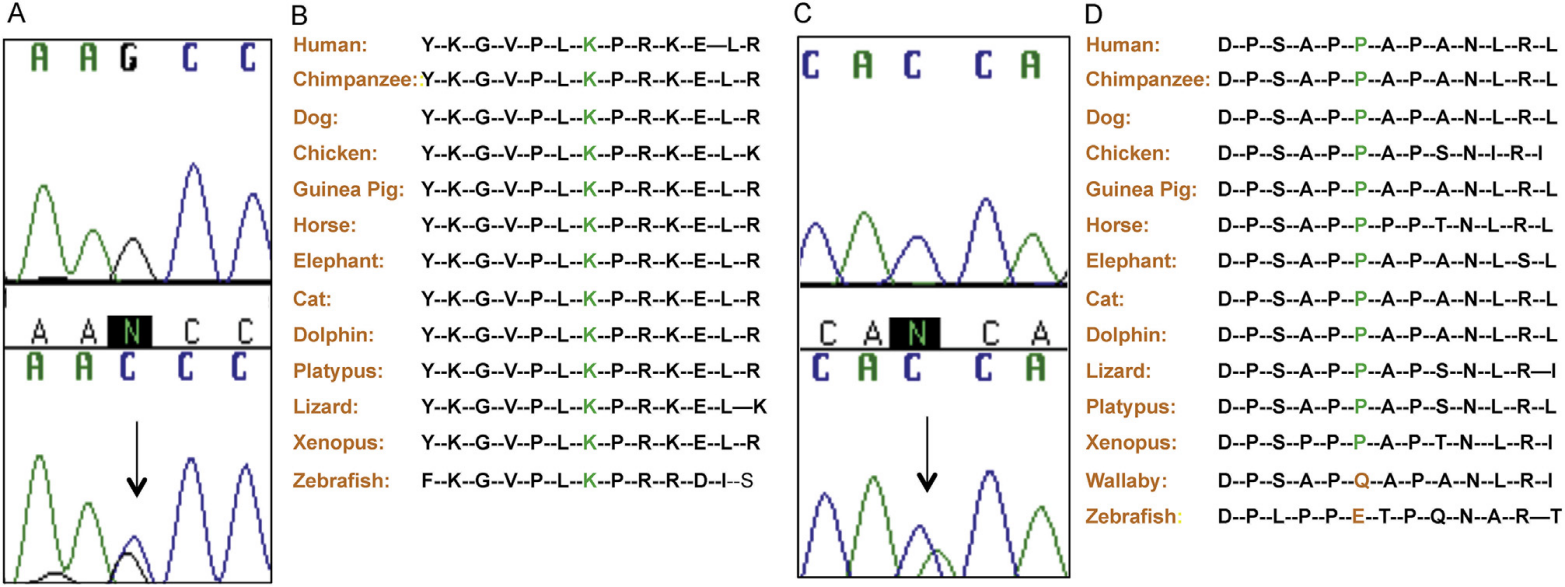
Electropherogram representation of two heterozygous variants found in *KAL1* and their conservation across multiple species. Variant c.555G > C, p.K185N (A; mutant sequence on bottom indicated by arrow) was detected in a female patient with septo-optic dysplasia, characterised by optic nerve and pituitary defects. The variant was highly conserved from mammals to zebrafish (B). Variant c.871C > A, p.P291T (C; mutant sequence on bottom indicated by arrow) was detected in two sisters with septo-optic dysplasia characterised by optic nerve and pituitary defects, as well as cavum septum pellucidum. The variant was highly conserved across mammals (D).

We detected two heterozygous *KAL1* sequence variants occurring at conserved alleles in three females from two unrelated families with SOD (Fig. 1); c.555G > C (p.K185N) identified in a patient with bilateral optic nerve hypoplasia, hypopituitarism and an ectopic posterior pituitary on magnetic resonance imaging (MRI), and c.871C > A (p.P291T), detected in two sisters with bilateral optic nerve hypoplasia, anterior pituitary hypoplasia and a cavum septum pellucidum. In both cases their mothers were unaffected heterozygous carriers. None of these patients harboured mutations in any other gene analysed and none of the 480 controls harboured either variant. However, despite their high interspecies conservation, both variants appear rarely (<1%) in the dbSNP, ESP and ExAC databases: p.K185N; 0.01%–0.07% in Caucasians, 0.0%–0.06% African Americans and p.P291T; 0.0%–0.09% in Caucasians, 0.0% African Americans. The sisters presenting the p.P291T variant were of African/Caucasian descent, and both variants have been predicted to be functionally deleterious by two separate algorithms (SIFT and Polyphen II). p.K185N has also been identified previously in a female with GnRH deficiency (Shaw et al. 2011).

#### 3.1.1 Patient 1 (c.555 G > C, p.K185N)

This female patient was the first child of unrelated Caucasian parents, born at term by forceps delivery, with a birth weight of 3.76 kg (+0.31SDS). She developed hypothermia and persisting hypoglycaemia on day 1 of life. A baseline screen at the time of hypoglycaemia (glucose 1.4 mmol/l) showed an undetectable cortisol (<0.9 μg/l) with an insufficient rise in adenocorticotrophic hormone (ACTH) of 5 ng/l (range 5–50 ng/l) suggesting ACTH deficiency, free T4 of 1 ng/dl (range 0.69–1.69 ng/dl) and a thyroid stimulating hormone (TSH) of 4.5 mU/l. Subsequent investigations revealed a free T4 in the low normal range of 0.89 ng/dl (range 0.69–1.69 ng/dl) suggesting evolving TSH deficiency, and the patient was treated with hydrocortisone and thyroxine. A pituitary MRI performed in the neonatal period revealed an ectopic posterior pituitary and bilateral optic nerve hypoplasia. At the age of 18 months her height was 81 cm (+0.19SDS), with a midparental height of −0.22SDS, and her weight was 13 kg (+1.58SDS). Testing with glucagon stimulation confirmed growth hormone (GH) (peak GH: 0.6 μg/l) and cortisol (basal and peak cortisol <0.9 μg/l) deficiencies; treatment with rhGH was then commenced. Visual acuity on the left was 6/6 with no perception of light on the right. She had normal development and no associated hearing or other systemic problems. By the age of 11.5 years there was no sign of puberty; she had a poor response to GnRH stimulation (peak luteinising hormone 0.6U/l, follicle stimulating hormone 2.7U/l) and puberty was induced at 11.9 years.

#### 3.1.2 Patients 2.1 and 2.2 (c.871C > A, p.P291T)

These siblings were born to unrelated parents of African and Caucasian origin. The eldest sibling was born at term with a birth weight of 2.32 kg (−2.85SDS). She had an uncomplicated neonatal period and first presented at the age of 6 months for assessment of nystagmus (left sided) and esotropia (right side). At that time she had a height of −1.34SDS with a weight of +0.23SDS and the MRI showed bilateral optic nerve hypoplasia, with a hypoplastic optic chiasm and a cavum septum pellucidum. Baseline investigations revealed a cortisol of 9.78 μg/dl, free T4 1.24 ng/dl (range 0.69–1.47 ng/dl) and TSH 3.3 mU/l (0.4–4.5 mU/l). She did not have dynamic testing of the hypothalamo-pituitary axis and received no hormonal replacements. She has visual impairment with no functional vision in the left eye and visual acuity 6/18 in the right eye.

The younger sibling was born at 37 weeks of gestation with a birth weight of +1.84SDS. She presented at the age of ten months with bilateral vertical nystagmus and inability to fix and follow. She had a height of 1.20SDS with a weight of 0.1SDS and MRI revealed bilateral optic nerve hypoplasia with a hypoplastic optic chiasm, thinning of the corpus callosum, and anterior pituitary hypoplasia with an absent posterior pituitary. She had a low peak GH to arginine stimulation (2.5 μg/l) and an adequate peak cortisol to synacthen (34 μg/dl), with normal thyroid function [free T4 1.24 ng/dl (range 0.69–1.47 ng/dl), TSH 1.4 mU/l (range 0.40–4.5 mU/l)]. She started treatment with rhGH and progressed well thereafter. There was no history of SOD in the family but their first cousin on mother's side has Moebius syndrome. None of the siblings had hearing defects or renal problems.

### 3.2 Protein-prediction modelling

**Fig. 2:**
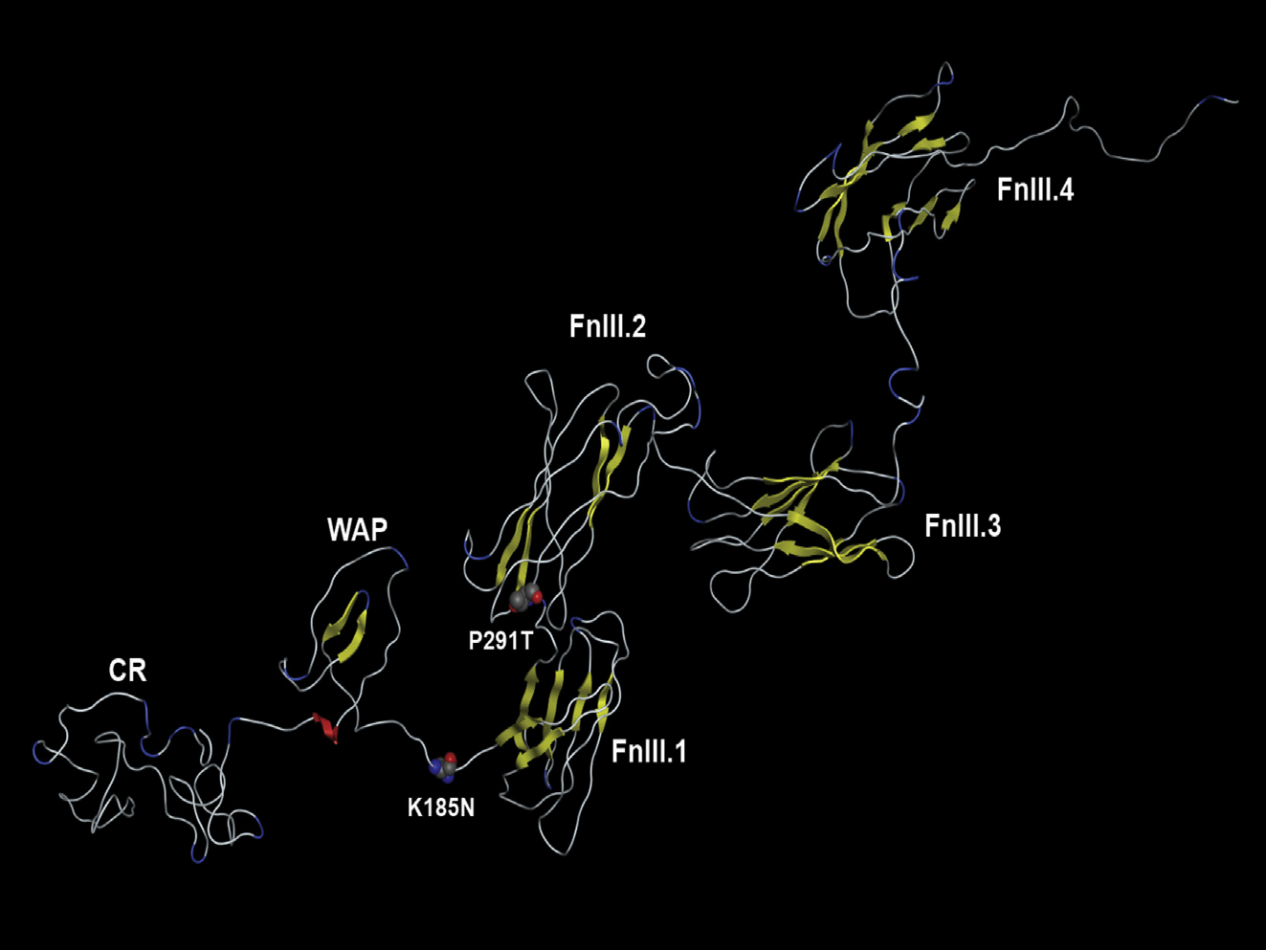
Ribbon representation of the six domain solution structure model of anosmin-1, N-terminal on left. Beta-strand ribbons in yellow, helix ribbon in red and turns in blue. The domains are labelled as follows: CR, cysteine rich region; WAP, whey acidic protein-like, FnIII, fibronectin type III. The p.K185N, located between WAP and FnIII.1, and p.P291T, located between FnIII.1 and FnIII.2, variations are indicated in space filling representation for the N and T residues. (For interpretation of the references to colour in this figure legend, the reader is referred to the web version of this article.)

To ascertain whether these rare variants detected in females with severe phenotypes would be ideal candidates for optimising a new functional assay for *KAL1* assessment, we conducted protein-prediction modelling. The p.K185N variant occurs in the loop between the WAP and first FnIII (FnIII.1) domains (Fig. 2). Since anosmin-1 seems to interact with FGFR1 within these domains (Hu et al. 2009), it is possible that the p.K185N variant affects this interaction. Alternatively, since the interaction of heparan sulfate, present in the extracellular matrix (ECM), is important for the localisation and binding of anosmin-1 to the ECM (Hu et al. 2004), the loss of the positively charged lysine at p.K185 may have implications for negatively charged HS binding.

The p.P291T variant occurs towards the beginning of the second FnIII domain (FNIII.2) (Fig. 2). The conformationally constrained proline being mutated to a more flexible threonine may alter the spatial disposition of the FnIII.1 and FnIII.2 domains. Alternatively, the p.P291T variation is close to a predicted HS proteoglycan binding site (S278–S293) on beta-strand G of FnIII.1 (Robertson et al. 2001) and may thus disrupt localisation and binding to the ECM. Subsequently, we decided that these variants were good candidates to continue functional assessment thereof.

### 3.3 Qualitative functional analysis

**Fig. 3:**
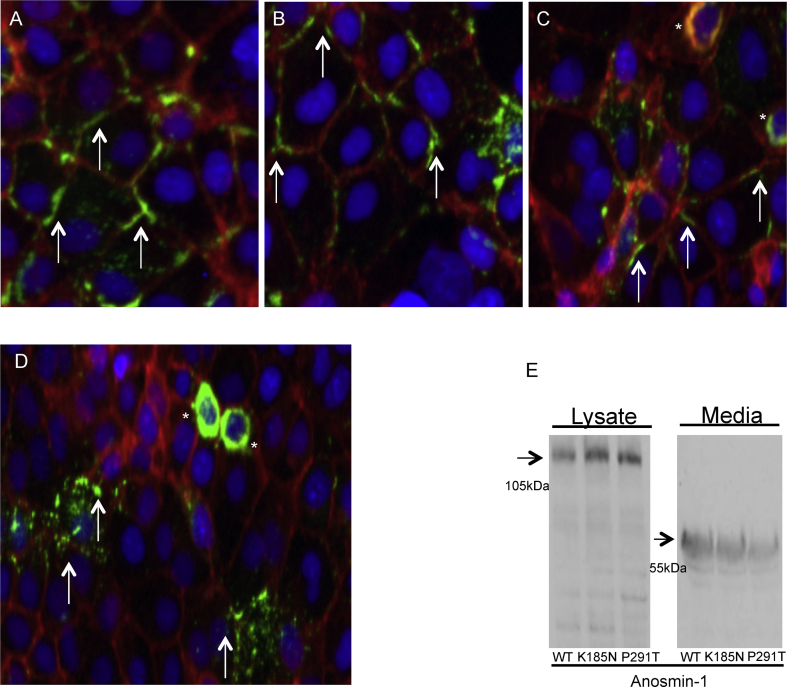
Qualitative functional analysis of anosmin-1 protein localisation/expression in transfected Cos7 cells. A-D, expression constructs encoding wt *KAL1* (A) or variant p.K185N or p.P291T proteins (B and C/D (low magnification) respectively) conjugated to GFP were transfected into Cos7 cells. Two days after transfection, cells were fixed for immunocytochemical analysis or lysed for protein extraction prior to western blot analysis (E). For the immunocytochemical stains, anosmin-1 (green), was co-localised with β-catenin of the adherens junction (red) to highlight cellular membranes. Cell nuclei were highlighted with DAPI (blue). Secreted anosmin-1 is indicated by arrows and retained protein is indicated by asterisks. E, western blot analysis was conducted on protein extracted from cell lysates or culture media. Bands corresponding to anosmin-1 are indicated by arrows. (For interpretation of the references to colour in this figure legend, the reader is referred to the web version of this article.)

Given the predicted changes in mutant protein function and particularly in conformation for the p.P291T variant, we assessed changes in their localisation relative to wt protein in Cos7 cells, using β-catenin as a cell-surface marker. Cells which had been transfected with wt anosmin-1-GFP, demonstrated co-localisation with β-catenin at the cell surface (Fig. 3A). Cellular localisation for the p.K185N-GFP variant appeared similar to wt (Fig. 3B). While p.P291T-GFP also appeared to be secreted from the cells, there was occasional evidence of its retention within the cytoplasm (Fig. 3C,D), an effect which was not observed with either wt or the p.K185N variant protein. Cellular retention of the p.P291T-GFP construct appeared to be qualitatively confirmed by Western blot analysis (Fig. 3E), however media extracts, but not cell lysates, demonstrated cleavage of the anosmin-1 product as previously described in the presence of serum (Regarli et al., 1996, Robertson et al., 2001, Hu et al., 2004). While this cleavage occurs after FnIII.2 and so downstream of our mutations, we considered it preferable to maintain a fully intact protein for quantitative functional analyses. For this reason serum was omitted from the media following transfection of the L6-myoblasts.

### 3.4 Quantitative functional analysis

**Fig. 4:**
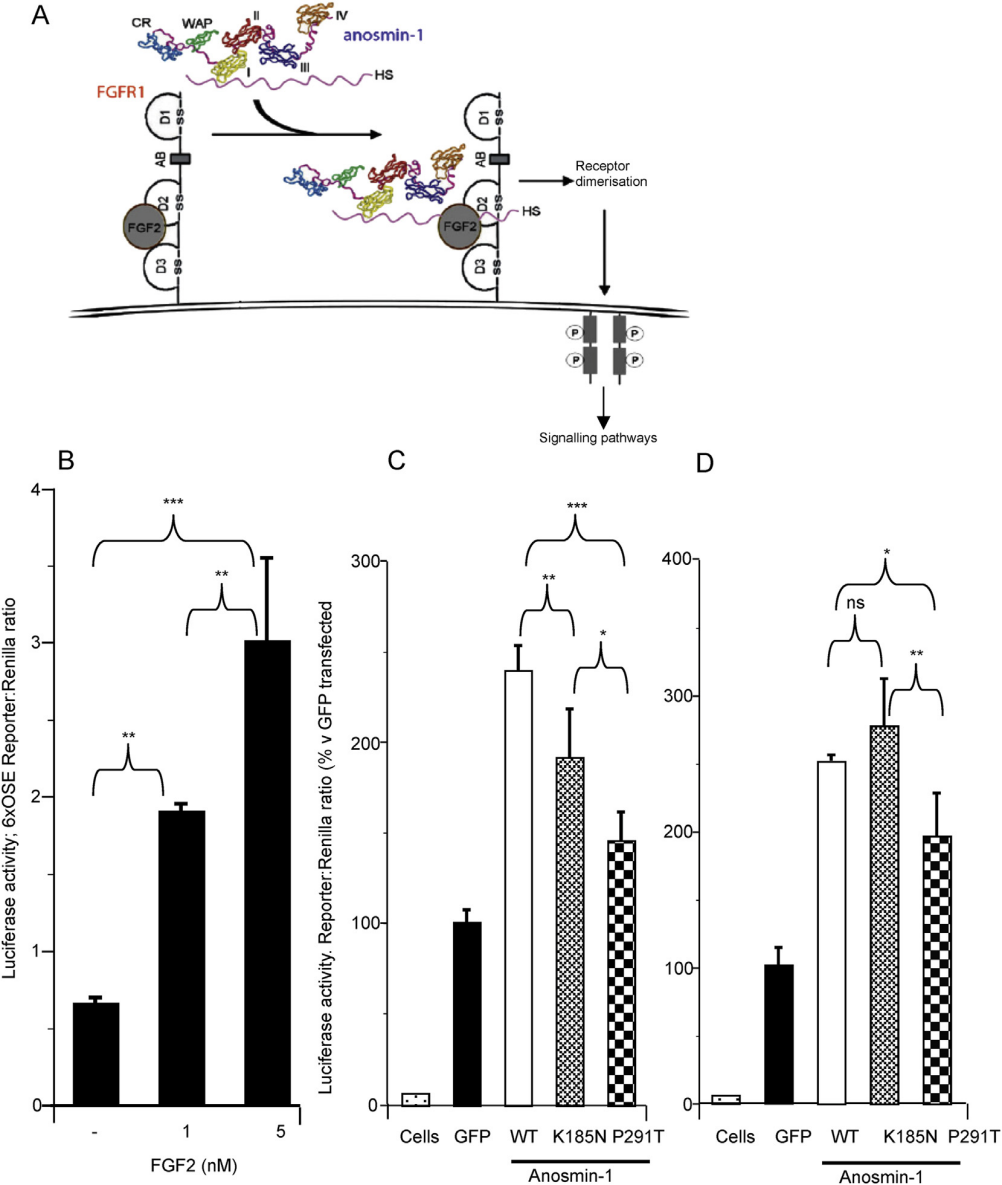
Quantitative functional analysis of anosmin-1 variants. A, Schematic representation of the FGFR pathway which our luciferase reporter vector (p6xOSE2-Luc) exploits. Binding of FGF2 ligand to FGFR1 stimulates dimerisation of the receptor and subsequent signalling cascades. These signals are enhanced through anosmin-1 binding to the receptor, which can be positively mediated by heparan sulfate. B, In order to validate our functional assay, FGFR1-expressing L6-myoblasts were transfected with our luciferase reporter and then treated with increasing doses of rhFGF2. C and D, FGFR1-expressing L6-myoblasts were then transfected with empty vector (labelled as GFP) or wt or variant *KAL1*-GFP conjugated constructs as well as our luciferase reporter and Renilla luciferase internal control. Cells were then treated with rhFGF2 with (D) or without (C) heparin (a type of heparan sulfate). The left column of each graph labelled ‘cells,’ were untransfected cells, still treated with 1 nM rhFGF2 ± 1 μg/ml heparin. All treatments were done in triplicate, with cultures repeated three times in total. Data are presented as mean ± SD. ns = not significant, * = p < 0.05, ** = p < 0.01, *** = p < 0.001. Note that panel A is adapted from Hu and Bouloux (2011).

Given the predicted deleterious effects of these detected variants and the occasional retention of the p.P291T variant, we proceeded with quantitative analyses of these changes. As previously described, our assay was designed to exploit the interaction of anosmin-1 with FGFR signalling (Fig. 4A). L6-myoblasts stably expressing *FGFR1* that had been transfected with the luciferase reporter only, were dose responsive to rhFGF2 (Fig. 4B). wt KAL1 treatment induced high luciferase readings (Fig. 4C), which were significantly greater than cells treated with either the p.K185N variant vector (21% reduction; p < 0.01) or the p.P291T variant (40% reduction; p < 0.001). The degree of functional impairment was also significantly different between the two variant constructs (p < 0.05).

Treatment with heparin in addition to rhFGF2 (Fig. 4D), abolished the difference in functional activity observed between wt and the p.K185N variation, but the p.P291T variant was still significantly compromised functionally compared to wt (p < 0.05) and p.K185N (p < 0.01).

### 3.5 Expression of KAL1 in the developing human embryo

**Fig. 5:**
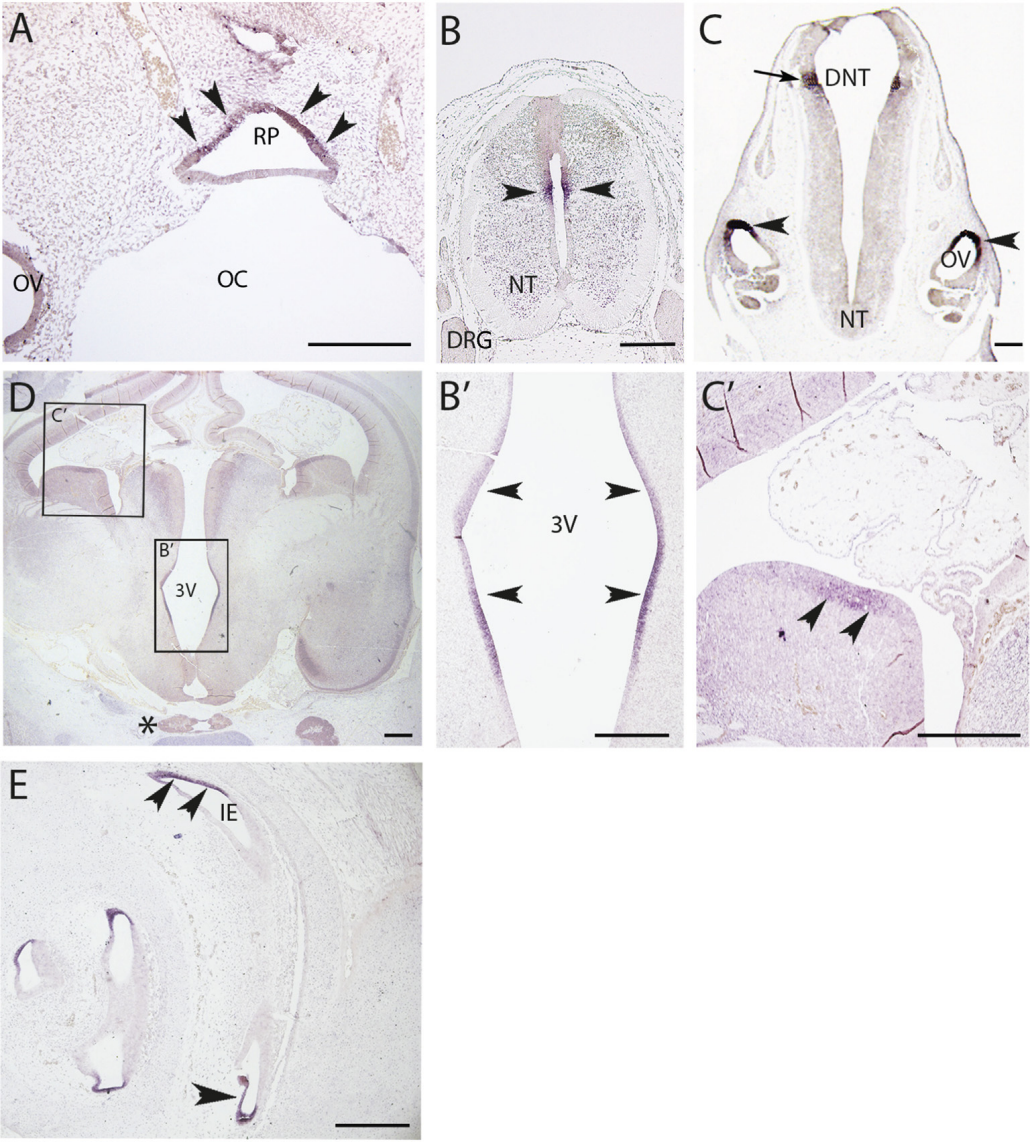
*In situ* hybridisation analysis of *KAL1* expression in the developing human embryo at 6 weeks (Carnegie stage 16) and 8 weeks (Carnegie stage 23) gestation. A, *KAL1* mRNA transcripts were observed transiently in the dorsal aspect of Rathke's pouch (primordial anterior pituitary gland) at 6 weeks (arrowheads). B, Expression was also observed in the developing neural tube (arrows heads in B and arrow in C). C, Strong localised expression was consistently observed in the dorsal aspect of the developing otic vesicles (OV, arrowhead). D, At week 8, *KAL1* mRNA transcripts were observed in the sulcus of the ventricular zone of the 3rd ventricle where the neural progenitors reside (labelled as 3V, arrowheads in B′), in the vicinity of the hypothalamus. No expression was detected in the pituitary (asterisk). B′ and C′ are enlarged images from D. C′ shows *KAL1* mRNA expression in the basal ganglion (arrowheads). E, *KAL1* was also observed in the developing inner ear (IE) that will form the cochlea (arrowheads). Abbreviations: 3V, third ventricle; DNT, dorsal neural tube; DRG, dorsal root ganglia; IE, inner ear; NT, neural tube; OC, oral cavity; OV, otic vesicle; RP, Rathke's pouch. Scale bars from A–D are 200 μm.

Given that we had identified functionally deleterious *KAL1* variants *in vitro* in three females with septo-optic dysplasia associated with pituitary phenotypes, we investigated a potential role for *KAL1* in human embryonic hypothalamo-pituitary development. *KAL1* gene expression at 6 weeks gestation (Carnegie stage 16) was detected in the dorsal aspect of the primordial anterior pituitary gland (Rathke's pouch) (Fig. 5A) as well as in the developing neural tube and dorsal aspects of the developing otic vesicles (Fig. 5B, C). By 8 weeks gestation (Carnegie stage 23), *KAL1* was observed at lower levels but restricted to the sulcus of the ventricular zone of the 3rd ventricle where neural progenitors reside in the hypothalamic region, and also in the basal ganglia (Fig. 5D, B′, C′). No pituitary expression was observed at this stage. However, expression was also seen in the inner ear canals that will form the cochlea (Fig. 5E).

## 4 Discussion

We have successfully applied a luciferase-reporter assay for novel quantitative assessment of rare variants/mutations in *KAL1*. Given that the two variants identified were rare, occurred at conserved residues, and were predicted to be deleterious *in silico*, we assessed their functional impact with our assay. The two variants detected herein in three females with SOD, p.K185N and p.P291T, were indeed deemed to be loss-of-function changes.

To investigate the variants, we used L6-myoblasts. This cell-line has been used for the study of FGFR1 signalling due to the intrinsically low to non-existent levels of expression of endogenous FGF ligands and receptors (Pitteloud et al. 2007). The cells used in this study had previously been stably-transfected with *FGFR1* (Hu et al. 2009). Both FGF ligands and receptors bind to HS and heparin (a heavily sulfated form of which is present in connective tissue mast cells); these cell surface proteoglycans are essential for the formation of the FGF-FGFR signalling complex (Mohammadi et al., 2005, Hu et al., 2009). However, other co-factors such as anosmin-1 are also known to interact with or regulate this pathway. This was postulated initially through the induction of neurite outgrowth and cytoskeletal rearrangements in a human embryonic gonadotrophin-releasing hormone (GnRH)/olfactory neuroblast (FNC-B4) cell-line via FGFR-dependent mechanisms, following recombinant anosmin-1 treatment (González-Martínez et al. 2004). Hu et al. (2009) subsequently confirmed this as a direct interaction of anosmin-1 with FGFR1-FGF2; enhanced by HS/heparin. The suitability of our model for this study was demonstrated through the dose response to rhFGF2 in luciferase reporter activity, and through discernible differences in receptor activity between wt and variant *KAL1* transfected cells.

The p.K185 residue lies between the WAP and first FnIII binding domains (González-Martínez et al. 2004). Although protein modelling predicted disrupted binding between the p.K185N variant and HS, treatment with heparin restored function in the presence of the variation. Potentially, by adding more HS to that already present endogenously, we overcame the functional deficit of the variant, as was also partially observed for p.P291T. Alternatively, our results were consistent with the known enhancement of interaction between anosmin-1 and FGF-FGFR signalling in the presence of HS as mentioned above (Hu et al. 2009).

The functional impact of the p.P291T variant was significantly greater than that observed with the p.K185N change (p < 0.05). Although the variation occurs just after the prime FGFR1/heparan sulfate-binding domain, it is hypothesised that it may disrupt final protein folding and conformation. Our qualitative studies suggest that the secretion of the protein may also have been disrupted, as demonstrated by the occasional retention of p.P291T-anosmin-1 within the cytoplasm in Cos7 cells.

One may then question how the loss-of-function variants detected herein fit within the broader context of KS and hypopituitarism/SOD. Our results, and that of a recently published finding which detected a missense *KAL1* variant in a male with hypopituitarism, support the known overlap in genotypes between KS and congenital hypopituitarism (McCabe et al., 2011b, Raivio et al., 2012, Vaaralahti et al., 2012, McCabe et al., 2013, Takagi et al., 2014). Due to the young age of the sisters with the p.P291T variant, it was not possible to test for KS associated phenotypes such as anosmia or hypogonadotrophic hypogonadism. The patient with the p.K185N variant however was non-responsive to GnRH and puberty was induced. Phenotypic manifestations in females due to mutations in *KAL1* may be possible given its localisation to the X-chromosome pseudoautosomal region (PAR). While most of the genes along one of the X-chromosomes are inactivated to ensure dosage compensation or equal gene expression between males and females, those within the PAR escape this process (Mangs and Morris, 2007, Shaw et al., 2011). This would appear to suggest that during fetal development, females would have double the gene dosage of *KAL1* compared to males. However, this may not be the case. On the Y-chromosome is a largely homologous *KAL1* pseudogene termed *KALP* (del Castillo et al., 1992, Montenegro et al., 2013), which is expressed in human embryonic stem cell lines. Study into the function of *KALP* has not been conducted to the best of our knowledge, but given the increasing evidence for a functional role for pseudogenes, a role for *KALP* could be worth exploring (Poliseno et al., 2010, Dhahbi et al., 2013, Pink and Carter, 2013).

However, care needs to be taken when interpreting our functional data in the clinical setting, as was recently demonstrated for *PROKR2*[p.L173R], where a homozygote was completely unaffected phenotypically (McCabe et al. 2013). The mothers of the variant-*KAL1* affected girls were unaffected carriers. This may indicate variable penetrance, but more likely, that other genetic or epigenetic factors may be implicated (Canto et al. 2009). Sequencing of other known KS and SOD genes revealed no other mutations; however given that genetic causes have been implicated in these disorders at a low incidence of ∼30% and ∼1% respectively (Dodé and Hardelin, 2010, Webb and Dattani, 2010), there is a large pool of contributory genes which have yet to be identified and which could be contributing to the phenotypes presented by our patients. Supporting this notion is the appearance of the variants in control databases (albeit rarely) and the relatively modest, albeit significant, decrease in mutant protein function detected herein in girls with such severe SOD and hypopituitarism phenotypes. The results presented herein are therefore suggestive that *KAL1* may be contributing to, rather than being causative of, the phenotypes observed in our patients.

In studies such as these, knockout or hypomorphic animal models are often employed alongside functional assays to consolidate or disprove the role of candidate genes in a given phenotype. As mentioned earlier, despite high conservation of *Kal1* from mammal to chick there is no mouse or rat homolog of the gene, hence difficulties in assessing its role in hypothalamo-pituitary development. Nonetheless, we attempted to assess a potential role for *KAL1* in this process. Our *in situ* hybridisation studies in 6-week (Carnegie stage 16) and 8-week (Carnegie stage 23) human embryos revealed early anterior pituitary and neural tube expression at 6 weeks, which had become restricted to the 3rd ventricle near the hypothalamus by 8 weeks. Mature GnRH-secreting neurons are located here, as are FGF-responsive cells. *Kal1a/b* knockdown in zebrafish results in disrupted GnRH and olfactory neuron maturation/migration, consistent with human phenotypes (Whitlock et al. 2005). In chick, concomitant with its expression in olfactory bulbs, forebrain and hypothalamus, Kal1 has been implicated in neural crest formation and shown to regulate Bmp5 and Fgf8 (Endo et al. 2012). Erroneous expression of Bmp5 in the chick leads to HPE and loss of midline structures (Golden et al. 1999), while in humans FGF8 has been implicated in HPE, SOD and Moebius syndrome, all in association with hypopituitarism (McCabe et al. 2011b). Notably, KAL1 has not been shown to regulate FGF8 in humans, but cumulatively, evidence from these models support our data which suggest a role for KAL1 in hypothalamo-pituitary development. Supporting this is the aforementioned paper published recently, which described hypopituitarism in a male with a missense *KAL1* variant (albeit of unknown functional significance), who also presented with combined pituitary hormone deficiency, right microphthalmia, renal aplasia, severe developmental delay and mild hearing impairment (Takagi et al. 2014). Interestingly, the detection of *KAL1* in the developing otic vesicles and inner ear canals in our *in situ* hybridisation is consistent with sensorineural deafness being attributed to *KAL1* deletions or missense mutations (Jansen et al., 2000, Söderlund et al., 2002).

In conclusion, we have developed a luciferase functional assay for novel application *in vitro* to demonstrate loss-of-function in rare variants in X-linked *KAL1* in three female patients with SOD and pituitary abnormalities. This assay may be suitable for assessing future or currently known variants/mutations in the *KAL1* gene. In the context of KS/SOD, the role of our detected variants in the presentation of the phenotypes herein is not entirely clear but they do extend the accepted overlap in genotypes/phenotypes between these disorders. The genotypic complexity of SOD suggests that other genes may be contributing to the phenotypes presented by our patients.

## Figures and Tables

**Table 1:** PCR-specific conditions used for *KAL1* amplification.

*KAL1* exon	Forward primer (5′-3′)	Reverse primer (5′-3′)	Product size (bp)	Annealing temp (^o^C)	MgCl^2+^ (mM)	DMSO +/−
1	GTTGCCTGGTCCTCAGCAGT	AACTTTGCGAGCCCAGGCTG	392	62	1.5	+
2	TCATTGGAAGGGAAGGACAG	ATTGGTGGAAACTGGGCATA	322	58	1.5	+/−
3	TTTGGTCCGCGTTCTGTAAT	TGACCCCACGTAAGCATAGTC	325	58	1.5	–
4	AAAGGTTTGGTGGGGAAAAA	CTGCCCCATGTCGAGTTAAT	526	58	1.5	–
5	AGGAGCAAATAGCTGAACTG	CGAGCACATTCGTTTGTATC	564	52	1.5	–
6	GCATTGGCTGATGGGTTAGT	AAAACAGCAAAGCCACCTGT	352	58	1.5	–
7	CCCCGTTTTCAGCTTTTCTA	GAAATGAATGGGAGGGAAGC	462	58	1.5	–
8	GCACCTGGCCTGAAGTTTAT	CTCCATTGTGCCTTGTTGTG	404	57	1.5	–
9	TGTGTTGTTCTCGCTGGAAG	CCTTAGTATTGATACTGTGGCTTGAC	363	58	1.5	–
10	ATCTCACCTCCTTTGGCTCA	CCATTCTGCTTTCCACTTCC	386	57	1.5	–
11	GGGAGTGTTTCACAATTGTCA	AGGCACTTTTGGTTTTCACG	469	58	1.5	–
12	GCCGCTGAAAATTCCTACAA	GCAAAAATAAGACTCAATAGTGCAG	424	57	1.5	+/−
13	AGAGAACCCCCACAAATGA	TTCCCTATCTCATAGAAACCTGT	438	58	1.5	+/−
14	CAGGGTCTCGTGCTTTTTCT	TGGAAGTGTGCATGTCTCGT	231	57	1.5	+
